# Shape Memory Alloy—Polymer Composites: Static and Fatigue Pullout Strength under Thermo-Mechanical Loading

**DOI:** 10.3390/ma15093216

**Published:** 2022-04-29

**Authors:** Stefano Rodinò, Elio M. Curcio, Danilo A. Renzo, Emanuele Sgambitterra, Pietro Magarò, Franco Furgiuele, Marco Brandizzi, Carmine Maletta

**Affiliations:** 1 Department of Mechanical Energy and Management Engineering, University of Calabria, 87036 Rende, CS, Italy; stefano.rodino@unical.it (S.R.); elio.curcio@unical.it (E.M.C.); danilo.renzo@unical.it (D.A.R.); emanuele.sgambitterra@unical.it (E.S.); pietro.magaro@unical.it (P.M.); franco.furgiuele@unical.it (F.F.); 2 Stellantis, Automotive Research & Advanced Engineering, 80038 Pomigliano d’Arco, NA, Italy; marco.brandizzi@crf.it

**Keywords:** shape memory alloys, smart composites, pull out strength, thermo-mechanical fatigue

## Abstract

This work was carried out within the context of an R&D project on morphable polymer matrix composites (PMC), actuated by shape memory alloys (SMA), to be used for active aerodynamic systems in automotives. Critical issues for SMA–polymer integration are analyzed that are mostly related to the limited strength of metal–polymer interfaces. To this aim, materials with suitable thermo-mechanical properties were first selected to avoid premature activation of SMA elements during polymer setting as well as to avoid polymer damage during thermal activation of SMAs. Nonstandard samples were manufactured for both static and fatigue pullout tests under thermo-mechanical loading, which are made of SMA wires embedded in cylindrical resin blocks. Fully coupled thermo-mechanical simulations, including a special constitutive model for SMAs, were also carried out to analyze the stress and temperature distribution in the SMA–polymer samples as obtained from the application of both mechanical loads and thermal activation of the SMA wires. The results highlighted the severe effects of SMA thermal activation on adhesion strength due to the large recovery forces and to the temperature increase at the metal–polymer interface. Samples exhibit a nominal pullout stress of around 940 MPa under static mechanical load, and a marked reduction to 280 MPa was captured under simultaneous application of thermal and mechanical loads. Furthermore, fatigue run-out of 5000 cycles was achieved, under the combination of thermal activation and mechanical loads, at a nominal stress of around 200 MPa. These results represent the main design limitations of SMA/PMC systems in terms of maximum allowable stresses during both static and cyclic actuation.

## 1. Introduction

Shape memory alloys (SMAs) are being used in an ever-increasing number of medical and industrial applications [1] due to their unique functional features and exceptional strain and force recovery capabilities, namely shape memory effect (SME) and pseudoelastic effect (PE). These properties are linked to a reversible solid–solid phase transition, the so-called thermoelastic martensite transformation (TMT), between two distinct crystal structures: the parent-body-centered cubic austenite (B2) and the product monoclinic martensite (B19′) [2]. Thermoelastic martensite transformation can be triggered by either temperature variations (SME) between the phase transition temperatures (TTs) or by mechanical stress (PE) between characteristic transformation stresses. Among the different types of SMAs, the binary nickel–titanium system (NiTi) exploits the best mechanical and functional performance, coupled with good corrosion resistance and biocompatibility, and its commercial success has seen an exceptional increasing trend in the last decades. Following the first widespread application of NiTi in medicine [3], where mainly PE is exploited, their industrial use is constantly growing in several sectors [4] from robotics [5,6] to automotive [7,8], aerospace [9,10,11] civil engineering [12,13], nuclear and heavy industry [14,15,16,17]. This favorable scenario is tremendously raising the interest of NiTi within the engineering community throughout the development of specific knowhow and design methods as well as by scouting new possible applications horizons.

Within this context, in the last few years, SMAs have been considered as unique materials for the realization of smart composites incorporating functional features of SMAs with advantageous structural properties of composites [18,19,20]. The combination of SMAs with polymer matrix composites (PMCs) offers the possibility of developing smart and lightweight components, with high load-bearing capabilities, exploiting the high stiffness and strength-to-weight ratio of PMCs and the unique shape and force recovery properties of SMAs. Possible applications of SMA/PMC systems span from active/adaptive vibration suppression [21,22] to high strength and self-healing components [22,23] and morphable structures and actuation [24,25].

One major issue for developing active/smart composites is the complex materials integration due to the large mismatch between physical and mechanical properties of metals and polymers. The large stress and temperatures occurring during SMA thermal activation could lead to early damage of the composite structure [26,27,28,29,30]. To this aim, material selection represents a critical issue for designing and manufacturing of SMA/PMC smart composites. The polymer matrix should satisfy mechanical and thermal constraints in terms of stiffness, strength and glass transition temperatures [31]. Conversely, chemical composition and processing methods of SMAs should be properly tuned to make the thermal activation compatible with thermo-mechanical properties of the polymer matrix. To overcome these difficulties, studies have been carried out in recent years to develop new design and manufacturing strategies for SMA-based polymer composites [18,31,32,33]. The effects of TTs of SMAs and glass transition temperature of polymers have also been analyzed in [24,34]. A low stiffness active composite was developed in [35,36], with SMA wires embedded in a silicone material, which can be used in medical applications (e.g., surgical and prosthetic devices). However, several technical issues related to SMA–polymer composites are still unsolved, mainly attributed to weak metal–polymer interfaces. Local stress distribution at the SMA–polymer interface can easily exceed the interfacial strength, especially if considering the limited thermal stability of polymers within the temperature range for SMA activation. This problem becomes even more complex during cyclic loading due to the unique fatigue [37,38,39] and fracture response of SMAs [40,41,42], which are linked to local stress and/or thermal induced transformation phenomena.

The aim of this investigation is to analyze the interfacial strength of SMA–polymer systems under both static and fatigue thermomechanical loading, through systematic studies involving both experiments and numerical simulations. For this purpose, SMA–polymer samples were manufactured by embedding commercial SMA wires in a thermoset polymer. An ad hoc testing rig was developed for testing the bi-material samples under complex thermo-mechanical loading conditions by combining mechanical loads with cyclic thermal activations. Multi-physics numerical models were also developed, including a special constitutive model for SMAs [43], for fully coupled thermo-mechanical analyses of SMA/polymer systems. Numerical models were aimed at analyzing the local stress and temperature distribution at the metal–polymer interface, under different thermo-mechanical loading conditions, and to understand the basic damage mechanisms. Results highlighted the main design limitations of SMA/PMC, due to the aforementioned issues, and they were allowed to identify possible viable alternatives for SMA integration in polymer matrix systems.

## 2. Materials and Methods

**Figure 1:**
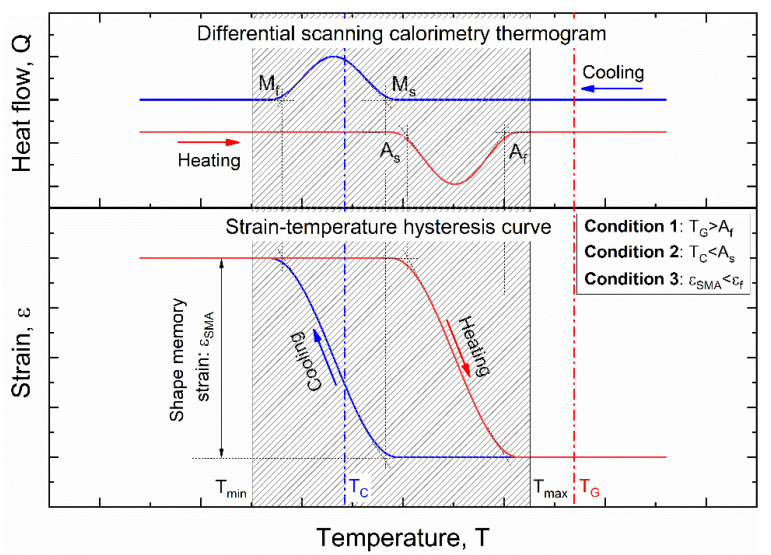
Schematic depiction of the transformation mechanisms in SMAs, in terms of DSC curve and strain–temperature hysteresis, and main thermo-mechanical constraints for SMA–polymer integration.

Both shape memory alloy and polymer materials for manufacturing of smart composites were preliminary selected based on strict thermo-mechanical constraints, as schematically shown in Figure 1. The figure reports a schematic depiction of the differential scanning calorimetry thermogram and thermal hysteresis of an SMA. A highlight of the phase transition temperatures (TTs) is also shown (namely austenite start (A_s_), austenite finish (A_f_), martensite start (M_s_) and martensite finish (M_f_)) together with the shape memory recovery strain (ε_SME_). Significant properties of polymers are also illustrated in the figure in terms of curing temperature (T_C_) and glass transition temperature (T_G_). Both manufacturing and operative constraints are identified as described below:

1. Condition #1: The polymer curing temperature (T_C_) must be below the SMA activation temperature (Austenite start, A_s_) to avoid early activation during polymer setting;
2. Condition #2: The polymer glass transition temperature (T_G_) must be higher than the SMA activation temperature (Austenite finish, A_f_) to prevent composite damage during thermal activation;
3. Condition #3: Mechanical strength of the polymer must be compatible with the stress–strain generated by shape memory recovery in SMA (ε_SMA_).

Based on the above conditions/constraints, a set of commercial materials was selected for SMA–polymer samples, as reported in Table 1. Other possible combinations of commercial materials that fit the above constraints could be identified, but it is out of the scope of this investigation. Significant thermomechanical properties of the selected SMA and polymer materials are reported in the following subsections.

### 2.1. Polymer Properties

The selected polymer (Epoxy crystal ng) is a two-component epoxy resin, with aminic hardener, having a good chemical stability that is mainly used for cold manufacturing processes. Polymerization occurs at room temperature without significant material shrinkage. This is a fundamental feature to avoid premature activation of the SMA wire during polymer setting (Condition #1 Figure 1). The main physical and thermo-mechanical properties of the resin, which are relevant for this study, are illustrated in Table 2.

### 2.2. SMA Thermomechanical Properties

**Figure 2:**
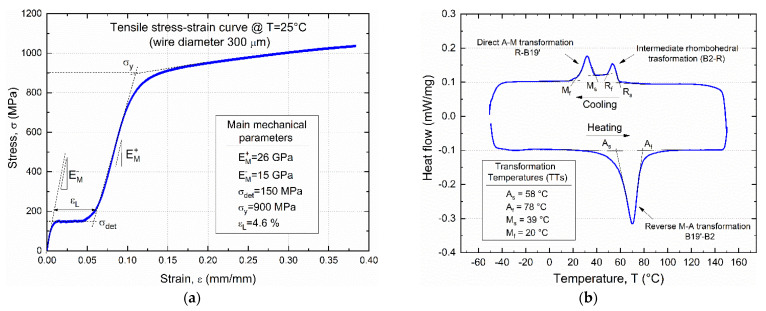
Thermomechanical properties of the selected SMA: (**a**) isothermal (T = 25 °C) stress–strain curve with main mechanical parameters; (**b**) differential scanning calorimetry thermogram with measured transformation temperatures (TTs).

Figure 2a illustrates the measured isothermal (T = 25 °C) stress–strain curve of the selected SMA as obtained from a strain-controlled tensile test of an SMA wire (d = 0.3 mm). The test was carried out after a complete thermal cycle by heating above A_f_ and cooling down below M_f_ to reset the material pre-strain, carried out by the manufacturer, and to obtain a full martensite structure. The main mechanical parameters of the SMA are also shown in the figure, namely Young’s modulus of twinned ($E_{M}^{-}$) and detwinned/oriented ($E_{M}^{+}$) martensite, detwinning/reorientation stress ($\sigma_{det}$) and strain ($\varepsilon_{L}$) and yield strength ($\sigma_{y}$).

Figure 2b shows the differential scanning calorimetry (DSC) thermogram of the selected SMA, as obtained from a complete heating cooling cycle in the range −50 ÷ 150 °C, together with the values of the transformation temperatures (TTs).

Evidence of intermediate rhombohedral phase (R phase) occurring during cooling from the austenite (B2-R) is shown on the DSC thermogram not directly exploited in this investigation. Thermal constraints (see previous section) are satisfied, as the measured value of the austenite start temperature (As = 58 °C) is higher than the curing temperature (T_C_ = 25 °C) of the polymer (condition #1), and the austenite finish (A_f_ = 78 °C) is lower than the glass transition temperature (T_G_~120 °C). However, actual activation temperature in SMAs can be higher than Af measured from DSC due to marked thermo-mechanical coupling effects, which are linked to both martensite pre-strain (ε_M_) and applied mechanical stress.

**Figure 3:**
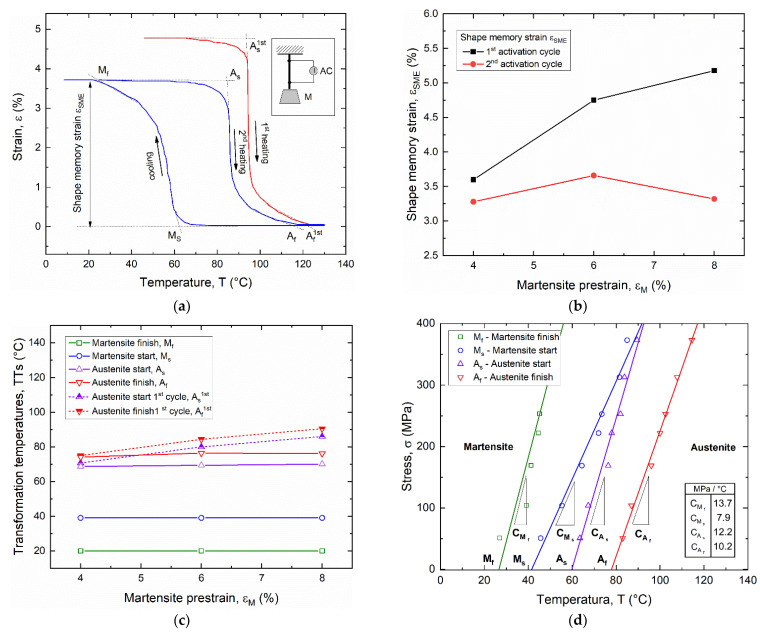
Shape memory effect in SMA wire: (**a**) strain vs. temperature thermal hysteresis for a martensite prestrain ε_M_ = 6% under an applied recovery stress σ = 150 MPa; (**b**) evolution of the shape memory recovery strain (ε_SME_) with martensite pre-strain (ε_M_); (**c**) evolution of the transformation temperatures with martensite pre-strain; (**d**) stress–temperature diagram.

To this aim, systematic studies were carried out to measure the shape recovery properties and related TTs under different loading conditions for different pre-strain values and applied recovery stress, as shown in Figure 3. Figure 3a reports the strain vs. temperature hysteresis loop of a wire, with a martensite pre-strain ε_M_ = 6%, subjected to the first thermal activation (T > A_f_) and subsequent complete thermal cycles in the range of 10 ÷ 130 °C under a constant stress σ = 150 MPa.

A marked decrease in the strain recovery (ε_SME_) between the first and second thermal activation is observed, together with negligible differences under subsequent thermal cycles. Figure 3b shows that strain recovery at the first activation cycle increases with martensite pre-strain, within the range 4–8%, whereas a non-monotonic trend is observed for the stabilized strain recovery, with a maximum value around 3.7% at ε_M_ = 6%.

A shift of the austenite TTs is also observed at the first thermal activation, due to martensite pre-strain, with a reset to the initial TTs at the second activation cycle. A similar behavior was observed for all investigated cases for martensite pre-strain (ε_M_) ranging from 4% to 8%, and the main results are summarized in Figure 3c. The shift of the austenite TTs increases almost linearly with martensite pre-strain up to a difference of about 20 °C at ε_M_ = 8%, corresponding to A_f_ around 90 °C.

Finally, Figure 3d reports the stress–temperature phase diagram of the alloy that is used to define the evolution of transformation temperatures with applied stress. This graph was built by a linear fit of TTs, as obtained from thermal hysteresis cycles (see Figure 3a), under different values of the applied stress in the range 50–375 MPa. The slopes of the curves (C_As_, C_Af_, C_Ms_, C_Mf_) represent the Clausius–Clapeyron constants for the four TTs of the material. Distinct values for the four TTs were captured, as reported in the figure, ranging between 7.9 and 13.7 MPa/°C. Furthermore, it is shown that the activation temperature (A_f_) rises to around 100 °C under an applied stress of about 200 MPa.

### 2.3. SMA–Polymer Sample Manufacturing

**Figure 4:**
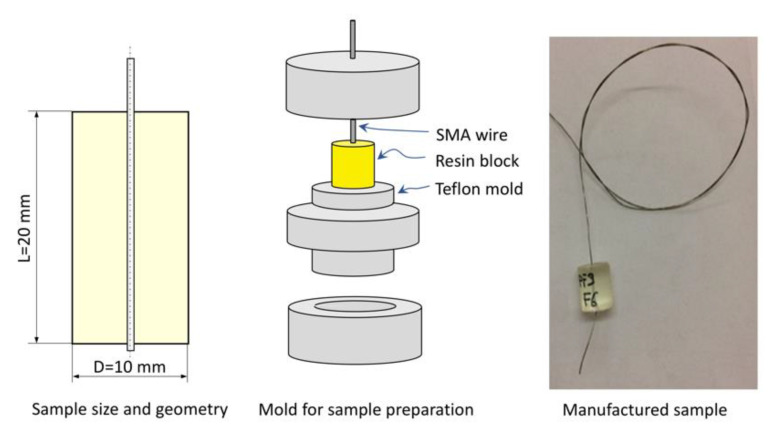
SMA–polymer sample for pullout tests: size, geometry and schematic of the manufacturing process.

SMA wires with a diameter (d) of 0.3 mm were used for SMA–polymer samples because they combine a good recovery force (F_rec_ around 20 N) with a large surface to volume ratio. The latter parameter is important, as it directedly affects the interfacial adhesion strength in SMA–polymer samples. As illustrated in Figure 4, samples are made of an SMA wire embedded at the center of a cylindrical polymer block (D = 10 mm, L = 20 mm). An ad hoc Teflon mold was used for sample preparation (see Figure 4). Polymer setting was carried out at room temperature (25 °C for 48 h) according to the manufacturer recommendations (see Table 2).

### 2.4. Static Pullout Tests

**Figure 5:**
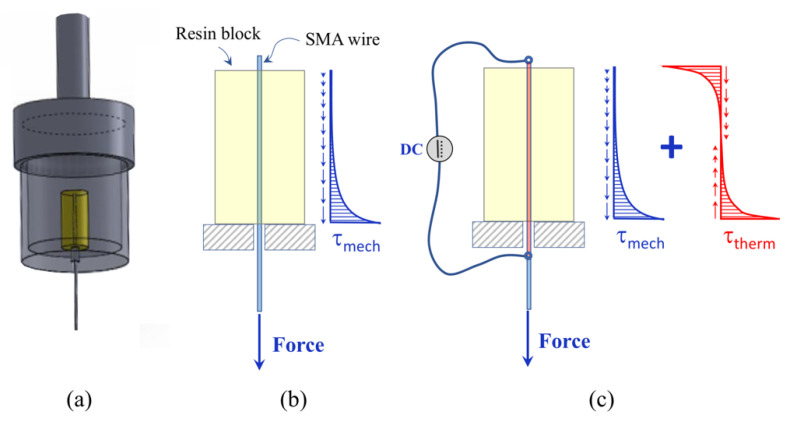
Schematic depiction of pullout tests: (**a**) loading tool, (**b**) mechanical loading (Load case #1) and (**c**) combined application of mechanical loading and thermal activation (Load case #2).

Static pullout tests were carried out to measure the adhesion strength of SMA–polymer samples by using a universal testing machine (E 10000, Instron, Norwood, MA, USA) and a special loading tool, as shown In Figure 5a. Displacement controlled tests were carried out under different thermo-mechanical loading conditions:

- Load case #1: Mechanical loading of as manufactured samples (Figure 5b);
- Load case #2: Combined mechanical and thermal loading of as manufactured samples (Figure 5c);
- Load case #3: Mechanical loading of samples after 1000 thermal activation cycles;

These load cases were selected to estimate the adhesion strength under both mechanical and thermal stresses, generated by SMA shape recovery, as well as to investigate on damage caused by cyclic SMA activations. Three different samples for each load cases were tested. Figure 5b show a schematic depiction of the mechanical loading condition together with the expected distribution of shear stresses at the interface, whereas Figure 5c is relative to the combined application of mechanical load and thermal recovery of the SMA wire.

### 2.5. Thermo-Mechanical Fatigue Tests

**Figure 6:**
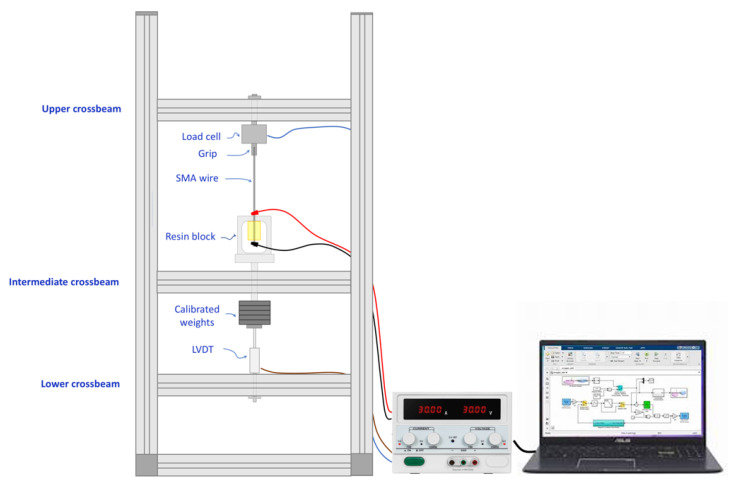
Testing rig for thermo-mechanical fatigue testing of SMA–polymer samples.

A special testing rig was designed and manufactured for fatigue testing of SMA–polymer samples under combined mechanical loading and thermal activation, as shown in Figure 6.

One free extremity of the SMA wire is gripped at the upper crossbeam of the frame that incorporates a load cell for direct measurement of the mechanical load. The resin block is positioned in a loading tool, whose design is similar to the one used for static tests (see Figure 5a). The connection rod of the tool is mounted in the central crossbeam by a low friction prismatic joint. Constant stresses can be applied by deadweights, and a linear variable displacement transducer (LVDT), mounted on the lower crossbeam of the frame, is used for measuring the displacement. Cyclic thermal loads can be applied by the Joule effect by a controlled electric current. The current is provided by a fully controllable electric power supplier (CPX400DP, Thurlby Thandar Instruments Ltd., Huntingdon, UK) whose cables are connected to the SMA wire in the near extremities of the resin block (see Figure 6). This setup allows for thermal activation of a limited length of the wire embedded in the resin block. A data acquisition system (QuantumX, Catman, Hottinger Brüel & Kjær, Marlborough, MA, USA) and a personal computer are used for real-time current control and data acquisition. Finally, the temperature of the SMA wire is captured by an infrared camera (A615, Teledyne FLIR LLC, Wilsonville, OR, USA) with a resolution of 640 × 480 pixels and a thermal sensitivity of 0.05 °C.

### 2.6. Numerical Modeling

**Figure 7:**
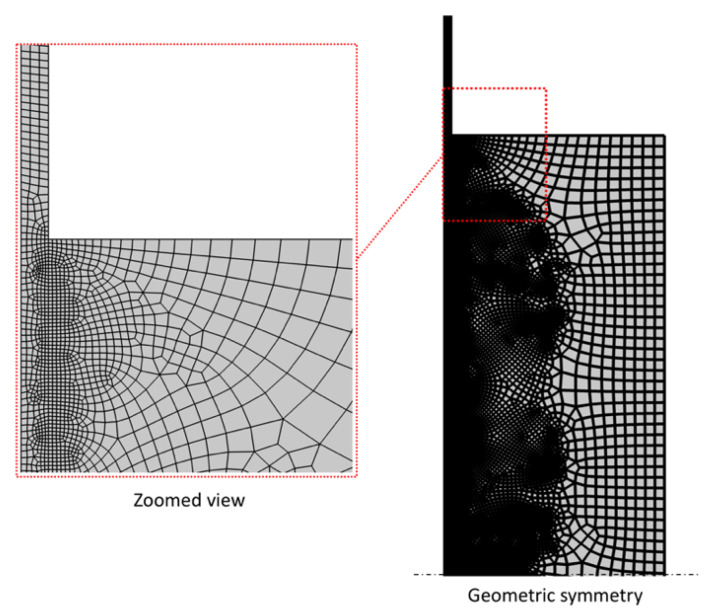
Finite element discretization of the SMA–polymer sample by 2D four-node axisymmetric quadrilateral elements.

Finite element simulations were carried out, by using a commercial software code (COMSOL Multiphysics^®^, 5.0, COMSOL Inc., Stockholm, Sweden) to analyze the stress and temperature distribution in the SMA–polymer sample under different thermo-mechanical loading conditions (see Figure 5). A 2D axisymmetric model was developed that is made of about 36 k four-node quadrilateral elements, with a refined mesh at the metal–polymer interface for an accurate simulation of the local thermo-mechanical interaction between the two materials (see Figure 7).

A special non-linear constitutive model for SMAs was adopted, based on the Lagoudas model [44] already implemented in COMSOL. The basic mechanical equation of the model is as follows:

(1) $$\bar{\sigma }=\bar{E}\left(\xi \right)\left[\left[{\bar{\varepsilon }}_{e}-{\bar{\varepsilon }}_{tr}-\bar{\alpha }\left(\xi \right)\left(T-T_{0}\right)\right]\right]$$

where $\bar{\sigma }$ is the stress tensor, ${\bar{\varepsilon }}_{e}$ and ${\bar{\varepsilon }}_{tr}$ are the elastic and transformation strain tensors, $T_{0}$ is the reference temperature, ξ is the martensite volume fraction,$\;\bar{E}\left(\xi \right)$ and $\bar{\alpha }$(ξ) are the elastic matrix the coefficient of thermal expansion (CTE) tensors whose coefficients are expressed as a function of the martensite volume fraction as follows:

(2) $$E\left(\xi \right)={\left[\xi /E_{M}+\left(1-\xi \right)/E_{A}\right]}^{-1}$$

(3) $$\alpha \left(\xi \right)={\xi \alpha }_{M}+\left(1-\xi \right)\alpha_{A}$$

where subscript *A* and *M* denote the austenite and martensite phases, respectively.

Furthermore, fully coupled electric–thermal–mechanical models were developed to simulate the thermal activation of the SMA wire by Joule effects. To this aim, heat transfer phenomena were set as conductive at the metal/polymer interface and convection with external environment.

The resin was modeled as an elastic material with Young’s modulus ($E_{R}$) defined as a linear function of the temperature:

(4) $$E_{R}\left(T\right)=E_{R0}-k\left(T-T_{0}\right)$$

where $E_{R0}$ is the Young’s modulus at the reference temperature $T_{0}$, which was set to 6.5 GPa at 25 °C, and k was set to 2.63 × 10^−2^ GPa/°C, corresponding to $E_{R}$ = 4 GPa at *T* = 120 °C. The CTE of the resin ($\alpha_{R}$) was assumed constant within the investigated temperature range. Table 3 summarizes the electric, thermal and mechanical properties of both SMA and resin used in this study.

The values of TTs used in the FE model (Table 3) are slightly different than the ones measured from DSC (Figure 2b), and they were obtained from a fine-tuning calibration process based on comparisons with experimentally measured strain–temperature hysteresis. This is to overcome basic model limitations that do not consider different slopes of the curves in the stress–temperature phase diagram (see Figure 3d).

**Figure 8:**
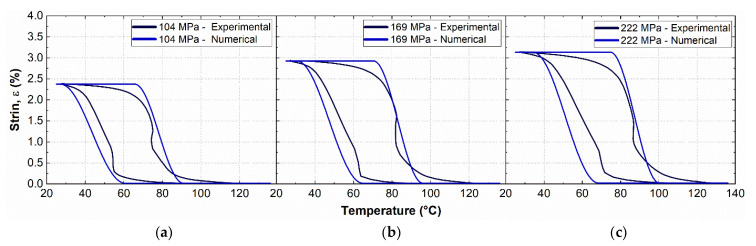
Comparison between experiments and numerical simulations of the strain–temperature hysteresis for a martensite pre-strain ε_M_ = 4% under different constant stress recovery conditions: (**a**) 104 MPa, (**b**) 169 MPa and (**c**) 222 MPa.

The calibration procedure is beyond the aim of this study, but a significant comparison between numerical and experimental results is shown in Figure 8 for the sake of completeness. Figure 8a–c shows the strain–temperature hysteretic response of the material, as obtained from a complete thermal activation cycle of the SMA (25–130 °C) subjected to a martensite pre-strain ε_M_ = 4% under different constant stress recovery conditions: 104 MPa, 169 MPa and 222 MPa.

The martensite pre-strain was directly set in the transformation strain tensor, due to the flexibility of the adopted FE package, in which thermal activation can be directly simulated without direct prior application of the mechanical pre-strain.

Both stationary and transient simulations were carried out to estimate the thermo-mechanical interaction between metal and polymer occurring under static and fatigue thermo-mechanical loading, respectively. This is of major concern due to the marked time-dependency of both conductive and convective heat transfer phenomena mainly affecting the temperature distribution of the sample under cyclic electric activations. To this aim, transient simulations were carried out in two steps:

*Step #1*: Transient electric–thermal simulations;

*Step #2*: Stationary thermo-mechanical simulations;

A simplified mesh was adopted for simulating transient phenomena due to electric–thermal coupling, consisting of about 5 k elements, to reduce the computational cost. The simulation time was set to 60 s, and an adaptive time step in the range of 0.001–0.1 s was adopted to improve the convergence of the model. The electrical load increment is based on discrete event step, to better simulate the behavior of the power supply. In particular, small time pulses are applied to the SMA material with a fixed time period.

The resulting maximum temperature distribution within the sample as obtained from transient electric–thermal simulation step, occurring at the end of the electric pulse, is subsequently applied to the full discretized model, and the resulting stress distribution is obtained by stationary thermo-mechanical coupled simulations.

## 3. Results and Discussions

### 3.1. Electric Activation of SMA Wires

**Figure 9:**
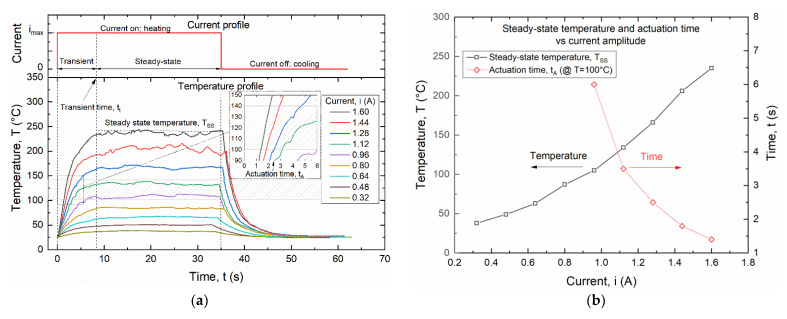
Temperature evolution of the SMA wire for a square current signal with a pulse duration of 35 s: (**a**) time evolution of the wire for different current amplitudes; (**b**) steady-state temperature (T_SS_) and actuation time (t_A_) versus current amplitude.

Figure 9 shows the effects of electric current on the temperature of the wire as obtained from IR measurements. In particular, Figure 9a shows the time evolution of the temperature for a square current signal with amplitude ranging from 0.32 to 1.6 A, and a pulse duration of 35 s. After a transient time, the temperature reaches a maximum steady state value T_SS_, corresponding to the thermodynamic equilibrium between heat generated by the Joule effect and heat exchange with the environment. Complete cooling down to room temperature is always obtained around 15 s after the pulse duration.

Figure 9a also shows a highlight of the temperature range for the thermal activation of the wire between the activation temperature of the SMA (T_A_ = 100 °C) and the glass transition temperature of the polymer. A zoomed view of the graph within this temperature region shows the approximate values of the activation time t_A_ defined as the time needed to reach the activation temperature T_A_.

Figure 9b summarizes results of the experiments in terms of steady state temperature (T_SS_) and actuation time (t_A_). It is shown that a minimum current of around 1 A is required to reach temperature T_A_, with an actuation time ranging from between 1.4 s at i = 1.6 A to 6 s at i = 0.96 A.

Additional experiments were carried out at higher electric currents, starting from the minimum current i = 1.15 A, with the aim of reducing the actuation time t_A_ and to speed up the fatigue experiments. However, steady-state conditions were not reached in these high current experiments, as the corresponding temperature T_SS_ would be extremely high for both polymer and SMA wire, as also shown in Figure 9a.

**Figure 10:**
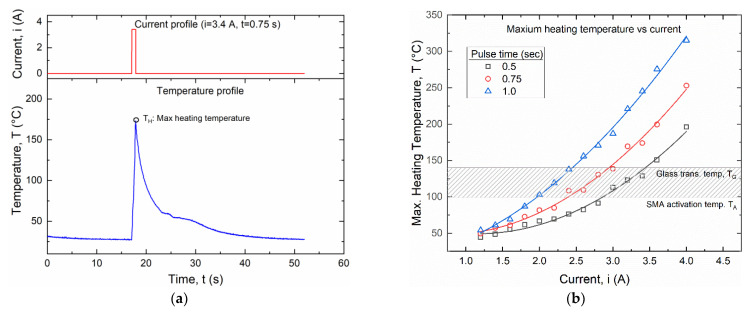
Temperature evolution of the SMA wire for a square current signal with small pulse durations: (**a**) time evolution of the temperature for i = 3.4 A and t = 0.75 s; (**b**) maximum heating temperature (T_H_) versus current amplitude (i) for pulse duration of 0.5, 0.75 and 1.0 s.

Square signals with small pulse duration (0.5 s, 0.75 s and 1 s) and current amplitude between 1.15 and 4 A were analyzed as shown in Figure 10.

Figure 10a shows the temperature evolution for a current of 3.4 A and a pulse duration of 0.75 s, representing the typical response for all investigated cases. Maximum temperature results as a function of the input current are summarized in Figure 10b together with a highlight of the temperature activation range. It is shown that a minimum current of 2 A can be used for a pulse time of 1.0 s and a maximum current of around 3.4 A can be applied for a pulse time of 0.5 s.

A current amplitude of 3 A and a pulse time of 0.75 s has been selected for the subsequent fatigue experiments according to these results.

### 3.2. Static Mechanical Strength

**Figure 11:**
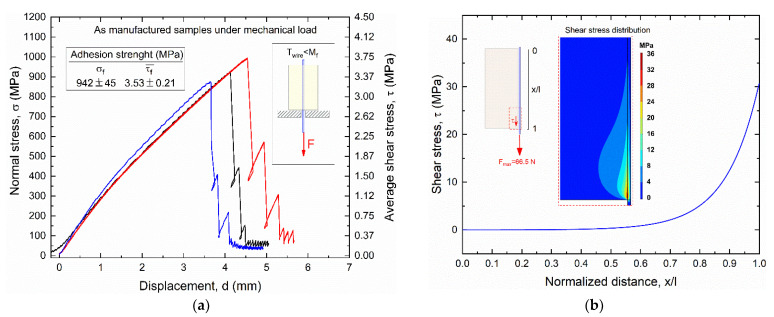
Results of _static_ pullout tests of as manufactured samples subjected to mechanical loading (load case #1): (**a**) experimental stress vs. displacement curves obtained from three different samples; (**b**) FE results of the shear stress distribution at the wire–resin interface along the contact length at the pullout force F_max_ = 66.5 N.

Figure 11 shows the results of static pullout tests of as manufactured SMA–polymer samples subjected to mechanical load (load case #1), as obtained from experimental tests (Figure 11a) and FE simulations (Figure 11b).

The left vertical axis in Figure 11a represents the actual normal stress in the cross section of the SMA wire, whereas the right axis is the average shear stress at the wire–polymer interface. This latter parameter is different than the maximum interface shear stress that occurs in the near extremity regions of the resin block, as clearly shown in Figure 11b.

Adhesion strength under axial loading is larger than the yield strength of the SMA wire (900 MPa) a normal stress to failure (σ_f_) around 940 MPa and an average shear stress to failure (τ_f_) of about 3.5 MPa, corresponding to a pullout force F = 66.5N. A little dispersion among the three samples was also captured with a coefficient of variation (σ/μ) around 5%. All samples exhibit an almost sharp drop beyond the peak load with a negligible residual strength after the first damage, and complete adhesive failure occurred at the metal–polymer interface. Figure 11b illustrates the shear stress distribution at the wire–resin interface along the contact length at the maximum pullout force F_max_ = 66.5 N, as obtained from the FE simulation.

A maximum shear stress τ_max_ of around 30 MPa is observed at the extremity of the bi-material sample where pullout force is applied (force application side) with a rapid drop to around 5 MPa in the first 5 mm (0.75 < x/l < 1), as also shown in the zoomed view with stress distribution contour maps in Figure 11b.

### 3.3. Static Thermo-Mechanical Strength

**Figure 12:**
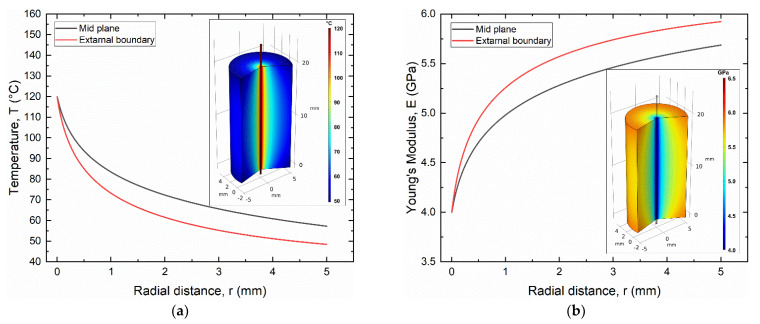
Finite element results of as manufactured samples subjected to mechanical loading and thermal activation of the SMA wire (load case #2): (**a**) temperature distribution in the resin block; (**b**) Evolution of the Young’s modulus in the resin block.

According to the electric activation results, thermal activation was carried out by a continuous electric current of 1.12 A, which allows for full activation of the SMA wire with a maximum steady state temperature (T_SS_) higher than 100 °C (T_A_) (see Figure 9). However, the effects of electric actuation of the SMA wire on the SMA–polymer sample were analyzed by fully coupled electric–thermal-mechanical FE simulations. Figure 12a,b shows the FE results of the temperature and Young’s modulus distribution in the SMA–polymer sample under steady state conditions, resulting from electric activation of the wire. The temperature in the resin block exhibits a rapid decrease from 120 °C at the SMA–polymer interface to about 40 °C at the external surface of the resin. However, effective temperature distribution under cyclic actuation is mainly affected by transient phenomena as analyzed in the following section. Figure 12b reports the evolution of the Young’s modulus of the polymer resulting from material heating, according to Equation (4). A reduction from about 6.0 GPa at the outer surface to 4 GPa at the wire interface is observed. This causes an increase in the deformation in the near interface region considered to play a role on the overall adhesion strength of the SMA–polymer sample.

**Figure 13:**
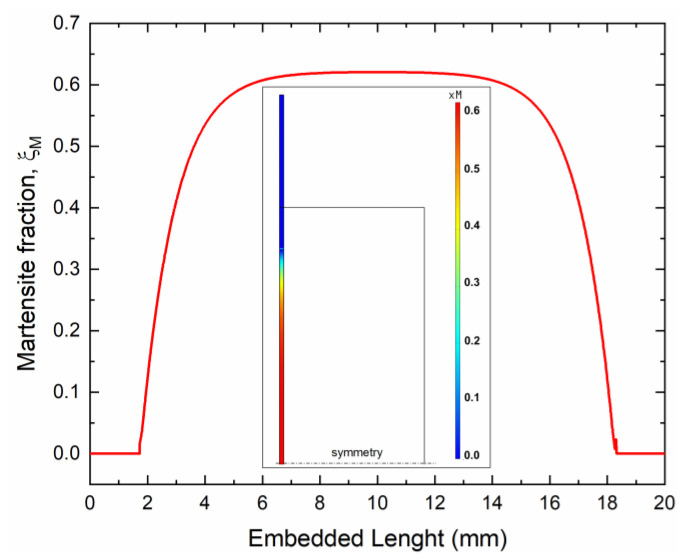
Martensite fraction in the SMA wire after thermal activation (i = 1.12 A) as obtained from finite element simulations.

Figure 13 shows FE results of the evolution of the martensite volume fraction (ξ_M_) in the SMA wire resulting from thermal activation. It is clearly shown that a fully austenitic structure is observed in the near free extremity of the sample (ξ_M_ = 0). On the contrary, partial transformations occur within the sample, leading to ξ_M_ = 0.6 in the middle section where only 40% of martensite is transformed in austenite, although the temperature is higher than the nominal A_f_ temperature. This is attributed to the normal stresses in the wire, resulting from SMA activation, which causes the actual A_f_ to rise according to the Clausius–Clapeyron relation, as also illustrated in the phase diagram of Figure 3d.

**Figure 14:**
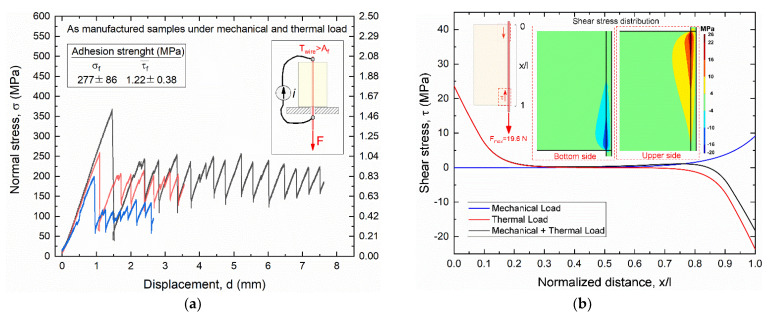
Results of static pullout tests of as manufactured samples subjected to mechanical loading and thermal activation of the SMA wire (load case #2): (**a**) experimental stress vs. displacement curves obtained from three different samples; (**b**) FE results of the shear stress distribution at the wire–resin interface along the contact length at the pullout force F_max_ = 19.6 N.

Figure 14 reports the results of the pullout experiments (Figure 14a) and FE simulations (Figure 14b).

Figure 14a shows a significant reduction in the applied mechanical stress to failure with respect to load case #1 with σ_f_ = 277 MPa and τ_f_ = 1.2 MPa corresponding to a pullout force F = 19.6 N. However, the total internal axial stress in the SMA sample is given by the combination of the applied mechanical stress and the recovery stress generated by SMA thermal activation, as schematically shown in Figure 4. The effects of the two loading conditions on the shear stress at the wire–resin interface are shown in the FE results of Figure 14b. It is shown that the SMA thermal activation generates a self-equilibrated and antisymmetric shear stress distribution with opposite signs at the two sides of the samples, with a maximum value of about 25 MPa. The maximum mechanical shear stress component is around 8 MPa, which is much lower than the load case #1 (30 MPa). Furthermore, the mechanical component at the bottom side of the sample (force application side), has an opposite direction with respect to the thermal one, resulting in a lower total shear stress with respect to the upper side (free end side) where only thermal stresses are present.

Nevertheless, these results demonstrate that the two load cases exhibit similar maximum shear stresses at failure around 30 MPa for load case #1 and 25 MPa for load case #2, although in the second case the external applied mechanical force is more than three times lower (66.5 N vs. 19.6 N).

However, normal stresses in the radial direction are also generated at the SMA–polymer interfaces that are expected to play an important role on pullout forces due to friction mechanisms. These stress components can be attributed to two distinct phenomena: (i) transversal mechanical strain components due to Poisson’s effects and (ii) differences in the thermal strains between SMA wire and polymer.

Regarding the Poisson’s effects, tensile stress in the wire causes a transversal diameter contraction, and similarly, compressive stress in the near interface zone of the polymer generates a hole contraction. Balance between these transversal strain components, which are due to different stresses and Poisson’s ratios in SMA and polymer, could lead to either normal tensile or compressive stresses at the interface. This is an even more complex phenomenon in SMAs due to the distinct Poisson’s ratios of the austenite and martensite phases.

Accurate measurements/estimation of the Poisson’s ratios of the two phases is extremely difficult due to the combination of thermal and stress-induced transformation phenomena, such that Poisson’s effects are intricately coupled with thermal and shape memory strains. However, it was found that martensite phase show values of the Poisson’s ratio in the range of 0.31–0.44 [28,44,45] that seem to be significantly lower than the austenite one, which would be in the range of 0.37–1.77 [28,44,45]. This is considered as an important effect when comparing pullout forces from load cases #1 and #2. In the case of combined application of mechanical stresses and thermal activation (case #2), the SMA wire is in austenite phase, and it would experience a larger diameter contraction with respect to the mechanical force only (case #1), resulting in a reduced pullout force. This effect was also observed in previous investigations [28] where pullout force under isothermal conditions in martensitic wires was found to be higher than in austenitic ones.

More intricate thermomechanical coupling effects occur in the present experiments due to the combination of mechanical loading and thermal activation of the SMA wire. Poisson’s effects are coupled with different thermal strains in SMA and polymer, which can be attributed to dissimilar coefficients of thermal expansion (CTE) and different temperature distributions in the two materials. From this standpoint, a larger expansion would be expected in the wire mainly due to the much higher temperature, resulting in a compressive normal stress and improving the pullout resistance. However, the total transversal effects in the SMA–polymer samples cannot be easily estimated due to simulation complexities and uncertainties about the evolution of material properties during stress and/or thermally induced phase transformation, such as in terms of CTEs and Poisson’s ratios.

However, the maximum normal stress is always higher than the detwinning stress of martensite (150 MPa) that is a critical condition for reversible actuation of the SMA–polymer system. Furthermore, a serrated trend of the curve is observed beyond the peak load that is attributed to stick-slip phenomena at the SMA–polymer interface, probably attributed to the increased polymer ductility at higher temperature.

### 3.4. Thermo-Mechanical Fatigue

Based on the thermal activation measurements described in Section 3.1, the cyclic actuation current was selected for thermo-mechanical fatigue tests with an electric current *i* = 3 A and a pulse duration of 0.75 s. This condition allows for complete thermal activation of the SMA wire (T_A_ > 100 °C) without excessive overheating that could cause polymer degradation.

**Figure 15:**
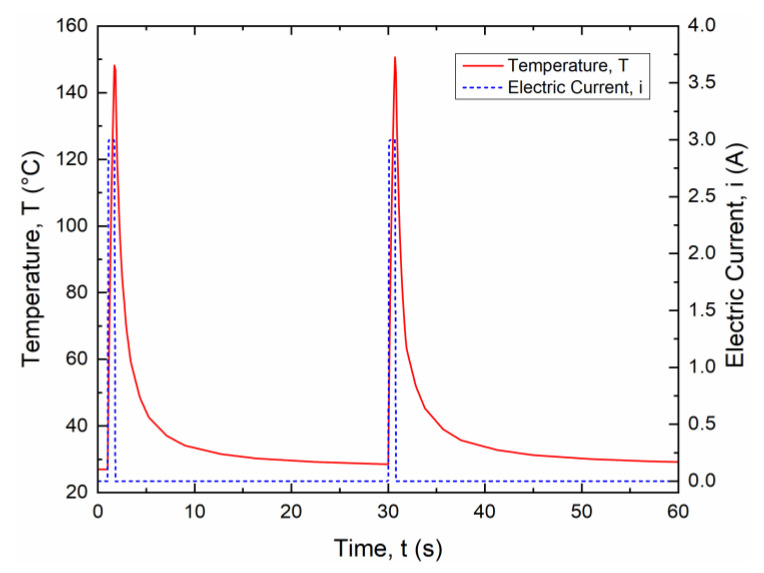
Time evolution of the temperature in the SMA wire for two subsequent thermal activation cycles (current—3 A, pulse time—0.75 s, period—30 s) as obtained from FE simulations.

The effects of thermal activation of the wire in the SMA–polymer sample were also analyzed by time-dependent FE simulations using a fully coupled electric–thermal–mechanical model. Figure 15 reports the time evolution of the temperature in the SMA wire for two subsequent thermal activation cycles (current 3 A, pulse time 0.75 s, period 30 s).

Maximum temperature (around 145 °C) is almost the same as the one measured by IR investigations on the SMA wire (Figure 10b). It is attributed to the negligible heat exchange with the resin block in the short pulse time (0.75 s). Similarly, a fast cooling is observed after the pulsed current with a sharp decrease in the first 5 s (to around 50 °C), as the resin block acts as a heat sink, and a subsequent slow decrease to room temperature is obtained after about 30 s. Accordingly, a period of 30 s was set for thermomechanical fatigue tests.

**Figure 16:**
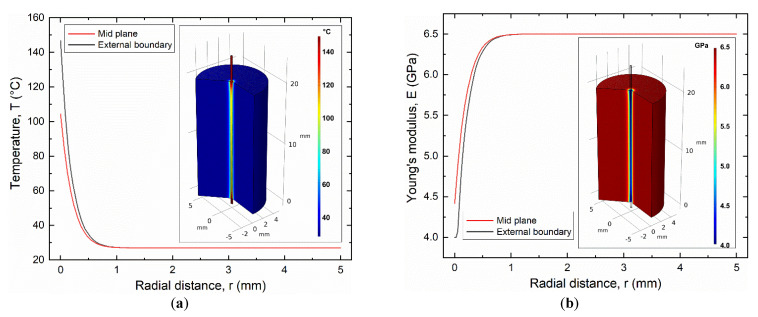
Effects of electric activation of the SMA wire by a pulse current (3 A, 0.75 s) as obtained from FE simulations: (**a**) temperature distribution and (**b**) Young’s modulus in the SMA–polymer sample.

Figure 16a,b reports the FE results of the temperature and Young’s modulus distribution in the resin block at the maximum current time (t = 0.75 s), respectively. Both figures show that thermal effects in the resin block are confined in a small radial distance from the SMA wire, where a sharp decrease to T < 100 °C is observed within a radial distance of around 100 μm and resin heating vanishes at around 800 μm. This result is significantly different with respect to the continuous current activation as reported in Figure 12, where generalized heating of the resin sample is observed with a temperature at the outer radius higher than 50 °C.

Thermo-mechanical fatigue tests were executed with a run out of 5000 cycles, and mechanical load was selected in the range 200–270 MPa. The lower value (200 MPa) is close to the minimum allowable stress for martensite detwinning (150 MPa), whereas the upper one (270 MPa) corresponds to the average value of the maximum static strength obtained from pullout tests in load case #2 (see Figure 12b).

**Figure 17:**
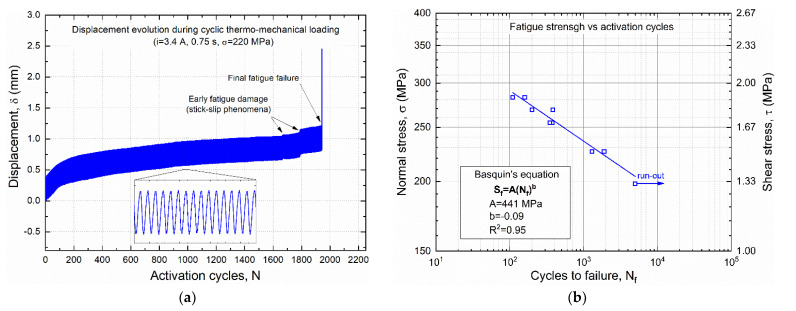
Thermo-mechanical fatigue of SMA–polymer samples: (**a**) evolution of the displacement vs. number of cycles under cyclic thermal activation (i = 3 A, t = 0.75 s) and a constant mechanical stress σ = 225; (**b**) Stress vs Cycles to failure (S--N) fatigue curve.

Figure 17a shows the characteristic evolution of displacement versus number of cycles, as directedly measured from the LVDT transducer of the testing rig (see Figure 6), which was obtained under a mechanical stress of 225 MPa. The curve shows an almost constant displacement amplitude associated with an increase in the average displacement, mainly attributed to creep-like phenomena and strain ratcheting of the SMA wire. These effects are more rapid in the first 100 cycles and then evolve almost linearly with further increases in the number of cycles. Fatigue damages are observed around 80–90% of fatigue life, which are attributed to stick-slip phenomena before complete failure. The latter phenomenon is exactly the same as for the static pullout tests, that is, the complete debonding between SMA wire and polymer block.

Figure 17b reports fatigue data obtained from all tested samples in the log-log S-N diagram. Data points are well fitted by a straight line (R^2^ = 0.95), and the corresponding parameters of the Basquin’s equation are also shown in the figure. Samples tested at σ = 200 MPa experienced run-out (5000 cycles), whereas failure always occurred at higher stress values, ranging from about 150 cycles at σ = 270 MPa to around 1500 cycles at σ = 235 MPa. These results make SMA–polymer integration compatible for the realization of smart composites, as 200 MPa is higher than the detwinning stress of SMA, which is required to obtain pre-strain at low temperature and shape recovery reversibility with heating and cooling cycles. However, great attention should be paid to the design task to not exceed this critical stress level given by the combination of the elastic bias of the deformed composite and the applied external load.

**Figure 18:**
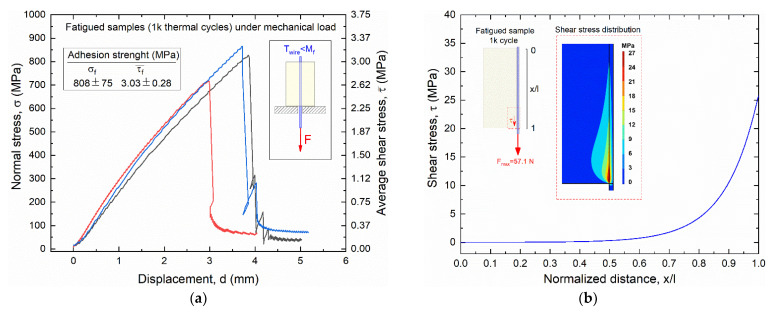
Stress vs. displacement curves obtained from pullout tests of SMA–polymer samples subjected to 1000 thermal activation cycles. Results of static pullout tests of samples subjected to 1000 thermal activation cycles: (**a**) experimental stress vs. displacement curves obtained from three different samples; (**b**) FE results of the shear stress distribution at the wire–resin interface along the contact length at the pullout force F_max_ = 57.0 N.

To better understand the role of cyclic thermal loads of the SMA–polymer samples, pullout tests were repeated after 1000 thermal activation cycles with no external stress applied. Results of pullout tests of such fatigued samples are reported in Figure 18 in terms of experimental stress vs. displacement curve (Figure 18a) and FE results (Figure 18b). Direct comparison with results obtained from static mechanical tests (see Figure 11) shows a reduction of the adhesion strength, with a decrease in the mean values from 942 to 808 MPa. Accordingly, a slightly lower maximum shear stress at the near extremity of the sample (25 MPa) is observed with respect to load case #1 (30 MPa), as shown in Figure 18b. These results confirm the important role of combined thermal activation and mechanical stress on static and fatigue damage of SMA–polymer samples attributed to both stresses generated by SMA activation and polymer degradation with increasing temperature.

## 4. Conclusions

A novel class smart composite with shape morphing capabilities, combining shape memory alloys (SMA) and polymer matrix composites (PMC), are being investigated for possible use in active aerodynamic components in automotives. SMA/PMC systems combine advantageous strength- and stiffness-to-weight ratios of polymer composites with the unique force and shape recovery capabilities of SMAs. The main issues for SMA–polymer integration were analyzed, which are mostly related to the limited strength of metal–polymer interfaces. To this aim, systematic tests of SMA–polymer samples were carried out under both complex static and fatigue thermomechanical loading conditions. Numerical simulations were also carried out, by using coupled electric–thermal–mechanical models for a better understanding of damage phenomena occurring under both mechanical load and SMA thermal activation. The main results can be summarized as follows:

- Static pullout strength of samples subjected to mechanical load (around 900 MPa) is remarkably higher than the martensite reorientation stress and is close to the maximum recoverable stress of SMA wires;
- Static pullout stress of the SMA–polymer samples are mainly unaffected by cyclic activation cycles (up to 1000). Maximum stress obtained from pullout tests is similar to that of manufactured samples;
- A marked reduction in pullout stress is observed under combined application of mechanical load and SMA thermal activation. This is attributed to the large interface stresses, which are due to both mechanical load and shape recovery in SMA, coupled with a reduction in polymer strength with increasing temperature;
- Fatigue strength corresponding to runout (5000 cycles) is still higher than the stress for martensite reorientation. This makes the SMA–polymer bi-material system suitable for repeated activations of morphable surfaces.

The obtained results will be used in future studies to design smart composite demonstrators with shape morphing capabilities. In particular, the interface strength obtained from this study from FE simulations of the sample failure conditions will be considered as main design parameters for smart composites.

## Figures and Tables

**Table 1:** Selected SMA and polymer materials for smart composite.

Material Type	Trade Name	Manufacturer
Shape memory alloy	SmartFlex	Saes Memry, Bethel, CT, USA
Polymer matrix	Epoxy crystal ng	Cores s.r.l., Parma, Italy

**Table 2:** Main physical properties of polymer.

Material Property	Value
Density, ρ	1.12 kg/dm^3^
Heat Deflection Temperature, HDT	64 °C
Glass transition temperature, T_G_	120 °C
Curing temperature, T_C_	25 °C
Curing time, t_C_	48 h
Compression strength, S_c_	60 MPa
Bending strength, S_b_	18 MPa
Tensile strength, S_t_	12 MPa
Young’s modulus, E	6.5 GPa at 25 °C 4.0 GPa at 120 °C
Poisson’s ratio, ν	0.4

**Table 3:** Main physical parameters of SMA and polymer used in the FE model.

FE Model Parameter		Value
Description	Symbol	
Transformation temperatures	M_f_	13 °C
	M_s_	50 °C
	A_s_	54 °C
	A_f_	81 °C
Clausius–Clapeyron constants	C_A_	13.9 MPa °C^−1^
	C_M_	12.2 MPa °C^−1^
Maximum recoverable strain	${ɛ}_{SME,\;max}$	0.061
Young’s moduli	E_A_	44 GPa
	E_M_	21 GPa
	E_R_ (@25 °C)	6.5 GPa
	E_R_ (@120 °C)	4 GPa
Poisson’s ratios	ν_A_	0.3
	ν_A_	0.3
	ν_R_	0.4
Density	δ_A_ = δ_Μ_	6450 Kg m^−3^
	δ_R_	1120 Kg m^−3^
Heat capacity constant pressure	C_pA_	600 J kg^−1^ K^−1^
	C_pM_	500 J kg^−1^ K^−1^
	C_pR_	1100 J kg^−1^ K^−1^
Thermal expansion coefficient	α_A_	11 × 10^−6^ K^−1^
	α_M_	7 × 10^−6^ K^−1^
	α_R_	26 × 10^−6^ K^−1^
Electrical resistivity	ρ_A_	86 × 10^−8^ Ω m
	ρ_Μ_	80 × 10^−8^ Ω m

## Data Availability

Data are available in the ARIA research project reports.
